# The “Materials Chemistry” Section of *Molecules*: A Multidisciplinary Environment for Materials-Based Researches

**DOI:** 10.3390/molecules25246035

**Published:** 2020-12-20

**Authors:** Giuseppe Cirillo

**Affiliations:** Department of Pharmacy, Health and Nutritional Sciences, University of Calabria, 87036 Rende (CS), Italy; giuseppe.cirillo@unical.it; Tel.: +39-0984493208

The “Materials Chemistry” Section of *Molecules* is an open access place for the dissemination of theoretical and experimental studies related to the chemical approaches to materials-based problems. Taking advantages from knowledge in chemistry, biotechnology, chemical engineering, physics, and materials science, the Section aims to become an interdisciplinary environment where the different expertise act synergistically to focus the progress in the field of creating and manipulating new materials. 

The Materials Chemistry Section benefits from the cooperation of 107 Editorial Board Members, responsible for the rigorous peer-review process and the high standard quality of the published papers. Their precious work allowed the impressive growth of the section, accounting for 567 published papers (seventh place among the different Molecules Sections). Authors and readers can take advantage of 117 Special Issues focusing on a specific area of materials chemistry and benefit from the contribution of recognized external Guest-editor or in-house Editorial Board Members. For celebrating the 25th anniversary, a Special Issue within the Section have been launched, together with a collection of the exclusive papers of the Editorial Board Members of the Section.

As all the fields of chemistry, materials chemistry is experiencing rapid growth by taking advantages from the developments in the synthetic and characterization procedures, as well as from the new technologies available at the micro- and nanoscale. I am honored and excited to be of service to the scientific community of this section, where a significant number of excellent contributions have been published within the last 2 years. 

The present editorial is aimed at summarizing some hot papers of the section, in order to provide readers with a vision of their relevance and contribution to the growth of materials science and technology. The selected papers cover studies related to both the synthesis and characterization of organic and inorganic materials, including biomaterials, nanomaterials, hybrid materials, core–shell materials, thin films, and self-assembling systems. 

Hydrogels, due to their fascinating physic and chemical properties, high hydrophilicity, swelling, micro-/nanosized pores, and softness partly mimicking the soft living tissues, are proposed for a plethora of applications in different fields of nano-/biomedicine, especially in tissue engineering. Cui et al. proposed gelatin methacryloyl hydrogels to construct multicellular cocultured 3D microtissues with high cell viability and improved functionality [1]. They fabricated cellular micromodules by photocross-linking of a mixture of gelatin methacryloyl and hepatocytes into hexagonal morphology with radial-type hole, which were subsequently assembled through local fluid-based micromanipulation. 

In biomedicine, hydrogels allowed the development of stimuli-responsive (or smart) delivery system able to fulfill specific therapeutic needs by controlling the release kinetics upon variation of an environmental stimulation. To this regard, Xu at al. reviewed the use of Schiff base linkages as click chemistry approach to synthesize smart hydrogels, i.e., at first, they classified and discussed the Schiff base chemistry highlighting the pH-responsive behavior and the possibility to modulate the biodegradation by selecting the appropriate nucleophilic agent to be reacted with the aldehyde functionalities, and then, epitomized the preparation and biomedical applications of hydrogels based on the Schiff reaction [2]. 

The deformation mechanics of chemically responsive hydrogels, including either the computational models for assessing the hydrogel free swelling or the techniques used for quantifying the gel deformation was reviewed by Fennel and Huyghe [3], while Vasile et al. [4] discussed the importance of the so-called hybrid materials, underlying that the terms hybrid hydrogels referred to the combination of either polymers with different origin (e.g., synthetic or natural) or polymeric materials with inorganic nanostructures. 

Different applications of hydrogels involved their use as a support for enzyme entrapment in the fabrication of solid biocatalysts. Amphiphilic-amino acid hydrogels composed of an aliphatic chain from myristic acid and l- and d-Phenylalanine were tested by Falcone et al. [5] for entrapment capabilities with horseradish peroxidase and α-amylase, showing improvement of enzyme stability and reusability. 

Composite systems in the form of nanomaterials have been also treated within the Materials Chemistry Section. Nanoparticle systems, indeed, by virtue of the high surface area to volume ratio and tuneable chemical and biological properties, offer a wide range of solutions for different therapeutic applications. Hong Wong et al. provided an exhaustive overview of polysaccharides and protein-based polymers investigated for nanosized drug delivery, discussing the development and clinical potential in their use as anticancer coadjuvants [6]. Janus et al., using glucose as a carbon source, developed carbon quantum dots functionalized with rhodamine derivatives as biomaterials to be applied in the area of cancer detection and treatment [7].

Nanoparticles are also attractive due to their antibacterial efficacy, as demonstrated in the study by Zhang et al., where upconversion composite nanoparticles NaYF4:Yb,Er containing Chlorin e6 with enhanced red light emission were designed for the efficient photodynamic treatment of periodontal disease (Figure 1) [8].

Apart than biomedical field, other applications of composite nanoparticles are photovoltaics and space missions. The improved photovoltaic performance of nanocomposite materials was demonstrated by Vázquez-López et al. by the synthesis of hybrid systems of SnO nanoparticles and conductive poly(3,4-ethylenedioxythiophene) doped with polystyrene sulfonate [9]. On the other hands, Winroth et al. reported the structure and performance of multifunctional polymer matrix composites composed of ultrahigh molecular weight polyethylene fiber reinforcement and a hydrogen-rich polybenzoxazine matrix, demonstrating clear advantages over benchmark materials in terms of combined structural and radiation-shielding performance [10].

The applications field of materials-based researches are almost limitless, as underlined by other interesting articles within our section.

With the aim of recognizing the future ahead of innovations in flame-retardant epoxy composites, Movahedifar et al. reviewed the reports on flame-retardant epoxy composites and classified them as a function of their flame retardancy performance by the use of the universal Flame Retardancy Index [11].

Bou Orm et al. proposed organic–inorganic hybrid nanotitania supports functionalized with a series of organophosphorus molecules holding different unsaturated groups, (including vinyl, phenyl, and naphtyl) for the efficient removal of polycyclic aromatic hydrocarbons pollutants through π-stacking complexation (Figure 2) [12].

Agrawal et al. addressed the need for the utilization of renewable energy sources for sustainable and environmentally friendly development of heating, ventilation, and air conditioning systems. They used nanostructured carbons (including carbon nanofibers and nanotubes) and the electrically insulating but thermally conducting hexagonal boron nitride (a structural analogue of graphite with alternating boron (B) and nitrogen (N) atoms instead of C atoms), to enhance the performance of organic phase change materials such as n-nonadecane and n-eicosane [13]. 

The interest of materials scientists in environmental problems was also highlighted in a review paper by Wang et al., where authors discussed the recent advances in the field of stable electrochemical energy storage systems (such as a supercapacitor, an ion battery, and a fuel cell) [14]. Finally, Saber et al. used explosive reactions to convert nonoptical alumina to series of new optical carbon nanotubes-based materials and tested the nanocomposite as a tool for using solar energy to purify water through photocatalytic degradation of green dyes [15].

These hot papers clearly demonstrated the impact of materials chemistry in everyday life. By hosting the recent advance in the field, the Materials Chemistry Section of *Molecules* aims to disseminate knowledges and promote cooperation between academia and industry in developing, improving, and finally scaling up new fascinating materials.

## Figures and Tables

**Figure 1:**
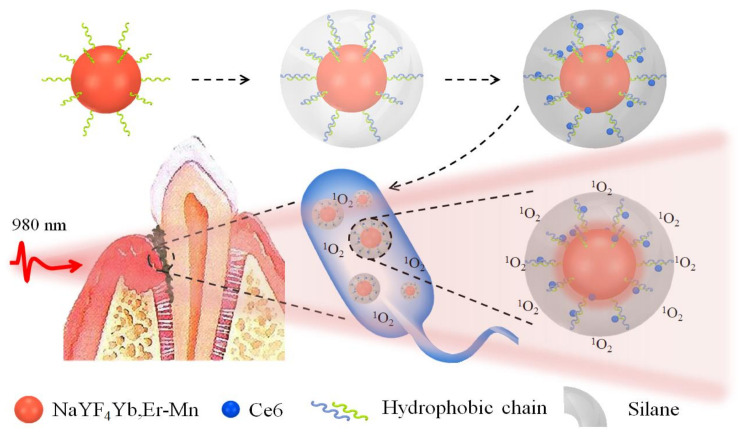
Composite nanoparticles for photodynamic treatment of periodontal disease [8].

**Figure 2:**
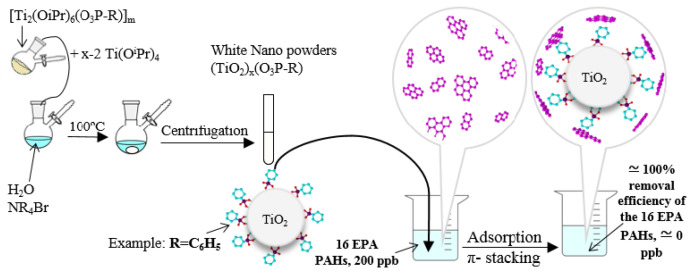
Composite nanoparticles for water purification [12].
